# Thread embedded into penile tissue over time as an unusual hair thread tourniquet injury to the penis: a case report

**DOI:** 10.1186/1752-1947-2-230

**Published:** 2008-07-16

**Authors:** Linus Ikechukwu Okeke

**Affiliations:** 1 Urology Division, Department of Surgery, College of Medicine, University of Ibadan, University College Hospital, PMB 5116, Ibadan, Nigeria

## Abstract

**Introduction:**

Hair thread tourniquet syndrome has been recognized since the 1960s. Since then, sporadic reports have appeared in the literature describing different degrees of strangulation and/or amputation of the penis caused by a hair thread being inadvertently tied around the penis.

**Case presentation:**

A 9-year-old boy presented with a 3-year history of hair thread tourniquet injury to his penis. Instead of the usual strangulation or amputation, the tourniquet had become embedded into the penile tissue, manifesting with exuberant granulation tissue and a tight urethral stricture. At surgery, the intact tourniquet was still in place, embedded in dense fibrous tissue and associated with a dense urethral fibrosis which measured about 2 cm long. The tourniquet was divided and removed, the fibrotic urethra excised and a distal penile pedicled skin flap used to perform a single-stage substitution urethroplasty. The patient has been voiding well for 28 months.

**Conclusion:**

This case is unusual and is the first report of its kind. It is also the first report of a hair thread tourniquet as the cause of pediatric penile injury in Nigeria.

## Introduction

Hair thread tourniquet syndrome has been recognized since the 1960s when a strangulating strand of hair was reported around the penis [1]. The patient usually presents acutely and responds well to removal of the tourniquet [2]. When there is a delay in presentation, partial amputation of the penis is often seen, with varying degrees of urethral transection [3].

We report the case of a 9-year-old boy who had a hair thread tourniquet on his penis for 3 years, but presented with only urethral stricture and granulation tissue and no strangulation or amputation of the penis.

## Case presentation

**Figure 1:**
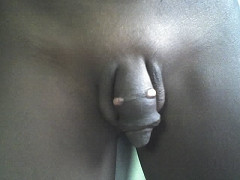
**The ring scar on the penis and the exuberant granulation on the dorsolateral aspects**. The skin distal to the scar appears normal.

**Figure 2:**
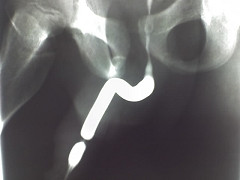
A retrograde urethrogram showing a tight ring urethral stricture in the middle third of the penile urethra.

A 9-year-old boy was seen at our urology outpatients' clinic in September 2005 with a 3-year history of straining at micturition and fleshy non-healing wounds located on the dorsolateral aspects of his penile shaft associated with minimal purulent discharge. The amount of discharge was not related to the act of voiding. He lived with his grandmother, who had tried topical applications of native medications without much improvement. On clinical examination, he had a ring scar located at the midshaft of his penis. The scar did not appear to be constrictive. There was no change in skin coloration or texture and no alteration of sensation distal to the scar. He also had minimal exuberant granulation tissue on the dorsolateral aspects of the scar associated with very minimal purulent discharge (Figure 1). There was a palpable dense periurethral induration beneath the scar. The bladder was not distended. His blood urea nitrogen, electrolytes and serum creatinine were normal. His retrograde urethrogram revealed a tight ring stricture in the middle third of his urethra (Figure 2).

**Figure 3:**
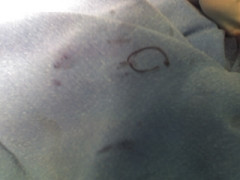
The black hair thread removed from the penis in the patient, placed on a piece of gauze swab.

At surgery under general anesthesia, a hemostatic tourniquet was applied to the root of his penis. A degloving incision was made in his old circumcision scar and the penile skin was dissected off the corporeal bodies towards the base of the penis. At the site of the ring scar, an intact black hair thread tourniquet was seen encased in dense fibrous tissue and binding the penile skin to the corporeal bodies. The hair thread tourniquet was divided and removed (Figure 3), and the penile skin sharply dissected off the corporeal bodies circumferentially at that location. A 2 cm urethral stricture was identified and excised, but when the urethral ends were temporarily pulled together, the penile shaft was severely bowed into a ventral chordee. A pedicled penile flap was therefore taken from the distal aspect of his penile skin and used for reconstructing the urethra over an indwelling urethral catheter after the method described by Quartey [4]. The procedure was completed by closing the circumcoronal wound, leaving a circumcision-like wound. A firm dressing was applied before the hemostatic tourniquet at the root of the penis was removed. His urethral catheter was removed 3 weeks postoperative. The patient's financial circumstances did not allow postoperative retrograde urethrography. He has since been voiding well and has been followed-up for 28 months.

## Discussion

Hair thread tourniquet syndrome usually occurs in infants and is presumed to be accidental [2,5]. However, Barton et al., in 1988, reported a case of hair thread tourniquet syndrome due to child abuse [6]. In the case reported here, a 9-year-old boy presented with a 3-year history, placing the incident most probably at age 6 years. He could not recollect the circumstances surrounding the tourniquet application, who had applied it and why it had been applied. He had not even been aware that there was a tourniquet on his penis.

The mechanism of injury in hair thread tourniquet is ischemia in the parts distal to the site of tourniquet application. Presentation in the acute state is usual, with swelling, erythema, circumferential constriction and distal edema [2] with little discomfort to the child [5]. This aspect of there being minimal discomfort to the child as well as his living with an aged grandmother whose initial recourse had been to native medication for 3 years may have contributed to his late presentation. If the tourniquet is not removed early, there may be progression to skin infection and ulceration, but removal of the constriction at this stage prevents long-term complications. In cases that are not recognized early, the dorsal neurovascular bundle may be transected, leading to the loss of sensation over the glans penis. The corpus spongiosum may become transected, leading to urethrocutaneous fistulation. The corpora carvanosa may also become transected, leading to partial or total amputation of the penis distal to the tourniquet [7-9].

It is worthy of note that even though the patient in the case presented here had his hair thread tourniquet for more than 3 years, there were no ischemic skin changes, no loss of sensation over the glans penis, and no urethrocutaneous fistulae. It is particularly unusual in that there was no degree of amputation of the part distal to the tourniquet. There was not even a discernible depression along the site of the tourniquet. The ultimate location of the hair thread was immediately subcutaneous, as if the tourniquet had 'sunk in' through the skin, embedded in fibrous tissue, with the intact dorsal neurovascular bundle running deeply in this. The granulation tissue associated with the ring scar reached only as far as the dense fibrous tissue surrounding the hair thread and did not seem to be related to the urethra.

A review of previously published reports from Nigeria did not reveal the hair thread as a cause of pediatric penile injuries in this region [10,11].

## Conclusion

The reason why this patient presented in this manner is not immediately apparent. It could be postulated that the hair thread tourniquet was not too tightly applied in the first place. The degree of ischemia was therefore not extreme and the associated tissue damage was minimal. Over the years, as the patient's penis grew, the intact tourniquet was gradually incorporated into the substance of the penis.

This case is peculiar and is the first report of its kind. It is also the first report from Nigeria of a hair thread tourniquet being the cause of pediatric penile injury.

## Competing interests

The author declares that he has no competing interests.

## Consent

Written informed consent was obtained from the patient's next-of-kin for publication of this case report and any accompanying images. A copy of the written consent is available for review by the Editor-in-Chief of this journal.
